# The Grand Challenge of Nephrology

**DOI:** 10.3389/fmed.2014.00028

**Published:** 2014-09-18

**Authors:** Howard Trachtman, Thomas Benzing, Sanja Sever, Raymond Clement Harris, Jochen Reiser

**Affiliations:** ^1^Department of Pediatrics, New York University Langone Medical Center, New York, NY, USA; ^2^Department II of Internal Medicine, Center for Molecular Medicine Cologne, Cologne Excellence Cluster on Cellular Stress Responses in Aging-Associated Diseases, Systems Biology of Aging Cologne, University of Cologne, Cologne, Germany; ^3^Division of Nephrology, Massachusetts General Hospital, Charlestown, MA, USA; ^4^Department of Medicine, Division of Nephrology and Hypertension, Vanderbilt University School of Medicine, Nashville, TN, USA; ^5^Department of Medicine, Rush University, Chicago, IL, USA

**Keywords:** nephrology, kidney, end stage kidney disease, basic science, clinical setting

## Pre-Modern History of Nephrology

The kidneys have always been an object of mystery and study from time immemorial. Confucius considered them to be one of the vital organs together with the brain and heart. The Talmud suggests that the kidneys are the seat of the soul, and the liturgy of the high holidays describes God as discerning what is in the heart and kidneys of man. Various psalms and passages from the Bible consider heart and kidneys as symbol of somatic and mental integrity (1). The Quran implies important functions are resting within the kidneys.

Richard Bright in 1827 was one of the first clinicians to recognize the connection between renal damage and illness. Although the label Bright’s disease is no longer in use, it is a testament to the astute acumen of this early 19th century physician. Although he is thought to have described a patient with post-infectious glomerulonephritis, the small, shrunken kidneys that are preserved in Guy’s Hospital in London do not provide a clue about the cause of the chronic disease. They only illustrate the consequences of renal scarring. Frederick Munk was the first to describe minimal change nephrotic syndrome in the 1910s and coined the term “lipoid nephrosis” for the disease called “dropsy” based on the appearance of the kidney tissue and urine. Although acute kidney injury was not a new entity, it was first diagnosed in living patients who suffered crush injury and rhabdomyolysis-induced kidney damage during the London Blitz in World War II. However, until the 1950s, there was very limited access to the laboratory tests required to establish a diagnosis of kidney disease, and there was no impetus to change things because there were no treatments for patients with renal disorders.

## Entry of Nephrology into Modern Medicine

In 1954, Merrill and Murray performed a kidney transplant between two identical twins and ushered nephrology into the modern age (2). There had been a number of pioneering physicians and engineers who were working on the development of an artificial system to replace kidney function when the organ fails. However, Merrill’s surgery represented a cure for CKD by implanting a new functioning kidney into a patient who had irreversible loss of organ function. He was fortunate to have access to an identical sibling and he could avoid the hazards of organ rejection due to immunologic disparity between the kidney donor and recipient. Over the next decade, the biology of tolerance and rejection was clarified by Peter Medawar leading to the discovery of immunosuppressive drugs. This has been an area of gradual improvement as safer and more effective drugs are isolated and incorporated into clinical practice. But it was Merrill who pointed the way toward the feasibility of correcting kidney failure and uremia. This spurred research into the pathogenesis of glomerular disease and clarification of the role of the immune system. Laboratory methods were introduced to enable routine measurement of BUN and creatinine in clinical practice. With all these developments, nephrology emerged as a vibrant subspecialty that combined exciting basic science and a genuine possibility of treating disease.

## The Golden Age of Nephrology

In the 1960s, clinicians finally took advantage of the work done by earlier investigators including Belding Scribner and began to implement hemodialysis for patients who had end stage kidney disease (ESKD). Originally, the procedure was very arduous and dangerous because the device was large and unwieldy and monitoring equipment was virtually non-existent. Early on, because dialysis was a scarce resource, committees were convened to decide which patients should be given access to this limited resource. However, this led to profound ethical dilemmas when these panels were forced to make life or death decisions regarding the care of individual patients. In response to this untenable situation, a law was passed by the US Congress in 1973 making any American citizen eligible for Medicare coverage of dialysis treatment. This represented the first time that an intervention to replace organ function became routinely available and that treatment for a specific disease was designated as a right or an economic entitlement. In response to this law, dialysis programs were opened throughout the US and the population of patients being treated with hemodialysis expanded dramatically. This number grew even further with the development of peritoneal dialysis, an important option especially in patients with severe atherosclerotic vascular disease or diabetes.

Nephrology practices and divisions grew in size as they reaped the benefits of the income generated by dialysis units. Nephrology was an attractive field for young physicians because it combined high level, intellectually challenging clinical work with sophisticated technical expertize. It offered the hope of ameliorating disease in patients who previously had languished and died due to lack of treatment. The excitement of dialysis and transplantation transformed the landscape of care for individuals with ESKD. Finally, advances in basic science such as perfusion of isolated tubules made it possible to address fundamental problems in renal physiology. Many departments of medicine were led by nephrologists and fellowship programs in nephrology successfully attracted the best and the brightest in medicine and pediatrics.

## The Technology Driven Age of Nephrology

However, there were problems looming large on the horizon as dialysis became the standard of care for ESKD. As the procedure became safer and more compact, it was applied to a wider range of patients. The nephrologists and legislators who passed the law making dialysis an entitlement originally thought that the procedure would be applied to a limited number of patients. However, over the next three decades, dialysis was implemented in an aging population with a wide range of co-morbid conditions including diabetes, atherosclerotic cardiovascular disease, and early dementia. The cost of the ESKD program grew exponentially provoking a strong federal backlash to limit spending on the dialysis. In addition, it became increasingly clear that dialysis was not curative and that the mortality associated with ESKD was delayed but not prevented. In part, the loss of enthusiasm for dialysis as a meaningful treatment option was paralleled by a lack of adequate federal support for kidney transplantation in a growing dialysis population. The gradual change in the patient profile, treatment fatigue with dialysis to manage patients with ESKD, and the growing perception that there were few meaningful therapeutic options led to a noticeable drop in enthusiasm among trainees for careers in nephrology and disillusion among established practitioners.

## Changing Place of Nephrology in Medical Care

While the incidence and prevalence of CKD and ESKD continue to increase, the discipline of nephrology faces serious challenges in the coming years related to manpower issues. In the US, trainees tend to no longer view nephrology as the most attractive subspecialty. This may have a number of reasons: (1) there has been an overall decrease in the number of medical students opting for internal medicine due to perceived concerns about lifestyle and compensation; (2) medical students and residents are often no longer exposed to the breadth of clinical nephrology or able to interact with nephrologists one-on-one in either preclinical or clinical settings; (3) there are negative perceptions about the practice of nephrology that relate to the long hours, relatively low compensation, focus on a chronically ill and complicated population of patients, and uncertainty about the future of the discipline. In this regard, changes in the “business of nephrology” in the past decades, with the centralization of providers to an increasingly smaller number of for-profit corporations and the rules, regulations, and payment limitations instituted by CMS, have led to a sense of loss of physician autonomy; and (4) many aspects of care that were historically under the purview of nephrology are being ceded to other disciplines, such as hypertension to cardiologists and fluid and electrolyte management to intensivists (3).

Although the number of US nephrology trainees increased from 711 to 911 during the period 2002–2009, the number of applicants for fellowship has been decreasing (hopefully), reaching a nadir in 2013, when 24% of the available positions were unfilled through the National Resident Matching Program. In addition, the percentage of applicants who are US medical graduates has steadily decreased, with 64% international medical graduates (IMGs) in 2013 (1, 4).

In addition to concerns about replenishing the nephrology workforce, there are also grave concerns about the future of nephrology research. In general, in the US, physician scientists are an increasingly rare and endangered breed, and this is especially true in nephrology, which has a strong and proud history of physician scientist-based discovery and scientific leadership. Furthermore, there are fewer opportunities for researchers in basic science departments to be exposed to cutting edge kidney-related research. In a recent analysis, Al-Awqati has noted that the percentage of nephrology-related papers published in The Journal of Clinical Investigation peaked in the late 1970s and early 1980s and have since “been declining over the past two decades in almost a linear manner” (5).

As with the causes for the drop in interest in a career in nephrology, there are numerous possible reasons for decreased interest in nephrology research. The debt burden of many medical school graduates is excessive, there is often a lack of exposure during training to appropriate role models or to kidney-related research, IMGs have more limited funding sources available for research training and must often opt for practice rather than pursuing careers as physician scientists, and there is general uncertainty about future job security as a researcher. The percentage of NIH dollars committed to kidney-related research has decreased steadily over the past decades. In 2012, even prior to the sequestration of 2013, the success rate for ultimately obtaining an RO1 for a kidney-related project had decreased to 17%. In 2011, the average age of a physician scientist receiving a first RO1 was 45 years (4). Even more disturbing, almost 30% of recipients of KO8 grants do not even apply for an initial RO1 (4).

Finally, it must be admitted that research in nephrology has not translated into the same advances in medical care as have been seen in many other areas of medical research. From 1966 to 2002, there were fewer RCTs in nephrology than any other medical subspecialty (6), and this disparity has persisted for the succeeding decade. Furthermore, between the years of 2002 and 2012, the FDA approved 37 urogenital-related indications, some of which were to previously marketed products with a new nephrology-related indication. Of these 37 approvals, the majority were not even for kidney-related drugs but addressed urologic issues such as bladder conditions or erectile dysfunction. For a comparison, during the same time period, there were 85 approvals for oncology-related indications, the vast majority of which represented new drugs resulting from insights gained from basic research (7).

## Current Changes in Nephrology: Clinical

With the arrival of the 21st century, it has become clear that nephrology trails most other medical subspecialties in the performance and completion of adequately powered clinical trials (8, 9). There are many factors that contribute to this issue, a number of which revolve around the nature of the patient population. These include the low prevalence of kidney disease in the general population, a misperception in lay community of the serious health implications of CKD, and a lack of novel agents with significant potential to favorably impact the course of common renal disorders. In addition, there has been a lack of standardization that has hindered accurate delineation of the epidemiology of common kidney problems. Finally, unlike oncology and specialized diseases such as adult respiratory distress syndrome, a stable renewable infrastructure has not been established to foster the timely performance of multicenter randomized clinical trials.

In recognition of these barriers to clinical research, the nephrology community has convened and begun to define standards for key clinical problems. The National Kidney Foundation has established a classification scheme for the definition of CKD that is divided into five stages. This has fostered studies into the incidence of CKD in different populations, delineation of the cardiovascular risk associated with CKD, and facilitated investigations of early biomarkers of declining kidney function. It is likely that this protocol will be revised to incorporate measurements of proteinuria to better refine diagnostic and prognostic implications of disease staging. In addition, the acute kidney injury network (AKIN) has drafted a scheme based on changes in the serum creatinine and urine output to categorize patients with acute kidney injury into stages with increasingly severe disease. The classification protocols have been revised and updated to reflect the unique features of specific patient cohorts such as children and neonates. But the overall effect has been to create a sense of organization and purpose to clinical research in AKI.

Glomerular diseases, in particular, are an important and growing cause of patient morbidity and mortality in all age groups. There have been few therapeutic advances in this area because of the rarity of the disorders and the need for multicenter collaborative efforts. In the last 5 years, the NIH-NIDDK has taken steps to rectify this problem by establishing prospective cohort studies of patients with incident (NEPTUNE) and prevalent (Cure GN) glomerular disease (10). These observational studies incorporate state-of-the-art technology in genomics, proteomics, metabolomics, and systems biology. It is anticipated that these efforts will yield an improved understanding of the pathophysiology of glomerular disease, provide better tools to define the nature and clinical course of specific glomerulopathies, and guide the design of therapeutics that target the underlying cause of each patient’s illness. This is likely to yield safer more effective treatments that can reliably prevent progression to ESKD. Close interaction with federal agencies, biotechnology companies, and the Food and Drug Administration will be needed to translate this hope for improvement into clinical reality.

In addition, to progress in the treatment of kidney disease, there is growing appreciation of the importance of prevention. This is underscored by the rising prevalence of CKD and high economic costs of treating the condition. Moreover, the problem is exaggerated in underdeveloped countries, where the lack of resources makes the burden of CKD especially heavy. A number of modifiable risk factors for CKD have been identified including non-traditional factors such as obesity, inflammation, and serum phosphate concentration (11). These factors are often present early in the disease course during young adulthood and can adversely impact on long-term outcomes (12). Thus, improved screening programs are being developed to enhance disease detection in high-risk populations and enable implementation of prevention strategies before irreversible injury ensues. Moreover, educational programs to increase awareness of effective strategies to prevent CKD are needed, and they should target all healthcare professionals including physicians, nurses, dieticians, and social workers. Much progress has been achieved in promoting these goals through international nephrology associations and patient advocacy groups.

## The Biomarker Dilemma

At present, the diagnosis of kidney disease is almost entirely based on the evaluation of renal biopsy. This invasive procedure – granted that it is rather safe – is inconvenient, takes time, and often fails to predict the course of the disease reliably. More recent advances in genetics and molecular biology are paving the way for a non-invasive diagnosis and improved prognostication. The discoveries of APOL1 in renal disease risk of African-Americans (13) or the existence of PLA2R antibodies in the diagnosis of Membranous Nephropathy (14) are among the recent highlights in our discipline. While we all embrace the discovery of such new markers, we must begin at a more rapid pace to integrate the associated tests into clinical trial design and also patient care. While this seems feasible, these tests are not yet uniformly available, and associated costs may become out of hand, hampering the building up of the momentum necessary to turn the flywheel for a new biomarker-driven diagnosis and prognosis for nephrology patients.

## The Necessity of Basic Scientists in the Clinical Setting

Impressive advances in basic science research have been made over the last 10–20 years that have improved our understanding of the pathogenesis of kidney diseases and that point the way to potential new therapies in the future. For example, preclinical studies suggest that renal injury may be reversible with implementation of treatment with angiotensin converting enzyme inhibitors or endothelin antagonists (15). Kidney-derived mesenchymal stem cells may represent a new approach to the treatment of acute kidney injury and CKD (16). Finally, discoveries about the role of the podocyte in the development of glomerular barrier dysfunction have been striking. These include identification of mutations in podocyte proteins that cause focal segmental glomerulosclerosis and the role of growth factors and circulating molecules in modulating podocyte integrity (17). These findings may pave the way to improved therapy of proteinuria and nephrotic syndrome.

The speed at which the field of nephrology will move forward with the development of novel therapeutics will depend heavily on the critical number of basic scientists deciphering the molecular mechanisms underlying the diverse etiologies of CKD. Although major funding agencies for research in the US such as the NIH are trying in a well-intentioned way to create connections between basic science and clinical research by urging basic scientists to explain how their research will ultimately impact human health, the reality is that such connections simply cannot be forced. Based on our cumulative experience, we suggest that the most productive connections between basic scientists and clinicians tend to form when a wealth of basic knowledge of the cellular and molecular processes acquired by basic scientists complement the wealth of knowledge of human physiology acquired by full-time physicians who treat kidney patients on a daily basis. In other words, a mutual interest must exist between the two parties for them to successfully work together.

One might wonder why basic scientists would not be highly interested in working with physicians and *vice versa*. From the perspective of basic scientists, it is not always apparent that the genes, proteins, or pathways that they are studying might have any relevance to a specific disease (or any diseases, for that matter). They may not even be necessarily driven to understand the mechanisms that underlie a disease, as they believe, and we agree that deciphering the secrets of nature should be sufficiently beneficial for humanity regardless of whether or not these discoveries ultimately lead to novel therapeutics. At the end of the day, not everything in research must be driven by the need to develop novel therapeutics or diagnostics.

From the point of view of the clinicians, their lack of familiarity with ever-changing basic science knowledge and concepts might prevent them from asking pertinent questions or from identifying scientists with relevant knowledge. Even if clinicians find the time in their already busy schedules to ponder the molecular mechanisms driving the diseases they encounter, they may still not be able to discern from the etiology of the CKD which pathways or molecules need to be studied. This problem is compounded by the fact that basic scientists and full-time clinicians often work in separate environments and rarely interact with one another. Even at teaching hospitals, clinicians traditionally reside within hospitals while scientists within their research laboratories. While clearly there is no single simple solution for this problem of workspace separation, there are a few things that could stimulate the formation of scientist–clinician collaborations.

Clinicians could reach out and approach scientists in their environment by offering collaborations – which, in practice, means providing human samples. Most scientists will at least try to listen and determine what can be done with those samples, with some scientists even coming up with very specific and creative ideas. Basic scientists, on the other hand, could attend meetings organized by and for clinicians. Listening to current clinical trials or controversies in the nephrology field would make it more obvious, where in a clinical setting, a scientist’s knowledge and understanding could best be utilized. Inviting more basic scientists to speak at such meetings would also prove productive for both sides. On a smaller scale, the same could be done more frequently at teaching hospitals or undergraduate universities. As editors of *the Nephrology section of Frontiers in Medicine*, we will try to do our part by creating a forum in which clinicians and basic scientists can be interactive in a collegial and collaborative manner. We hope to do this by enlisting a significant number of basic scientists to serve as reviewers and also encouraging them to submit their latest findings to the journal. We invite clinicians to do likewise in order to facilitate an open and dynamic intermix of basic scientists and clinicians and foster an exciting dialog. The journal will be fully committed to a fair and highly qualified review of all submitted research reports. In order to ensure a comprehensive assessment by reviewers who have competency in the topic under consideration, priority will be given to clinical scientists for the assessment of clinical studies while basic scientists will take the lead in the evaluation of preclinical reports. However, input from both sides of the investigative spectrum will be enlisted for all submissions in order to give each manuscript an open hearing and to ensure highest quality standards and utmost rigor.

## The Future Begins Now

Technology and consumer industry operate according to the principle of instant gratification. Under those standards, the world of medicine appears to be a clear outlier. The path from a promising discovery in the laboratory to an effective treatment often takes a decade or more. It may often be abruptly halted close to the goal because of new safety concerns or unanticipated adverse effects.

But through this process of trial and error, progress and setbacks, and finally sustained progress, insights are steadily growing. Advances that are changing the course of nephrology are beginning to be transparent to all who are involved in nephrology research and patient care. In order to maintain the accelerating pace of nephrology developments and to build momentum, we have to continue to seek for new ways to assess normal and pathological kidney function.

## Change is Difficult

Nephrology is a discipline that shares features with another prominent discipline, namely neurology. Both are characterized by a high fund of knowledge amongst the specialists but a difficulty to translate knowledge into clinical actions due to the lack of new medications or other technology of precision medicine. This is a huge problem that ultimately feeds back to our trainees who consider other career paths that may appear more exciting. Oncology has undoubtedly attracted many trainees because exciting new drugs are being developed in this area. What does that mean for our scientists or scientists who study the kidney and its related systems? We must embrace translational medicine and we must allow for molecules and novel drug targets to be further evaluated by industry even in the presence of some knowledge gaps. This is clearly a shortcoming in our field that only we can overcome – let us be brave.

## Conflict of Interest Statement

The authors declare that the research was conducted in the absence of any commercial or financial relationships that could be construed as a potential conflict of interest.
